# Hyperactivity of ON-Type Retinal Ganglion Cells in Streptozotocin-Induced Diabetic Mice

**DOI:** 10.1371/journal.pone.0076049

**Published:** 2013-09-19

**Authors:** Jun Yu, Lu Wang, Shi-Jun Weng, Xiong-Li Yang, Dao-Qi Zhang, Yong-Mei Zhong

**Affiliations:** 1 Institute of Neurobiology, Institutes of Brain Science and State Key Laboratory of Medical Neurobiology, Fudan University, Shanghai, China; 2 Eye Research Institute, Oakland University, Rochester, Michigan, United States of America; Dalhousie University, Canada

## Abstract

Impairment of visual function has been detected in the early stage of diabetes but the underlying neural mechanisms involved are largely unknown. Morphological and functional alterations of retinal ganglion cells, the final output neurons of the vertebrate retina, are thought to be the major cause of visual defects in diabetes but direct evidence to support this notion is limited. In this study we investigated functional changes of retinal ganglion cells in a type 1-like diabetic mouse model. Our results demonstrated that the spontaneous spiking activity of ON-type retinal ganglion cells was increased in streptozotocin-diabetic mice after 3 to 4 months of diabetes. At this stage of diabetes, no apoptotic signals or cell loss were detected in the ganglion cell layer of the retina, suggesting that the functional alterations in ganglion cells occur prior to massive ganglion cell apoptosis. Furthermore, we found that the increased activity of ON-type ganglion cells was mainly a result of reduced inhibitory signaling to the cells in diabetes. This novel mechanism provides insight into how visual function is impaired in diabetic retinopathy.

## Introduction

Diabetes is a serious and potentially life-threatening disease in humans. A major complication of diabetes is diabetic retinopathy, a current leading cause of blindness in adults [1]–[3]. Diabetic retinopathy is clinically diagnosed by the onset of vascular disorders, which has led to the general assumption that it is solely a microvascular disease [4]–[6]. However, recent studies indicate that this complication is also a neurodegenerative disease because both neuronal death in the retina and decreased visual functioning have been detected prior to vascular complications [5]–[21]. Unfortunately, the neural mechanisms involved in diabetes-induced early visual defects are poorly understood.

Early visual defects in diabetes appear to be a result of an impairment of the neural retina. A fundamental organization of the neural retina is that some cells respond to light increments (ON cells), whereas others are activated by light decrements (OFF cells) [22]. This segregation begins in the retina where the output ganglion cells can be divided into ON and OFF subtypes based on their responses to light. Retinal ganglion cells receive and integrate information from photoreceptors via bipolar cells and send the information to the visual centers of the brain. Ganglion cells are the most-studied retinal neurons with respect to the effect of diabetes [6]; ganglion cell degeneration has been reported in both diabetic patients as well as in animal models of diabetes [6], [17], [19], [21], [23]–[25]. In particular, a recent study has shown the structural remodeling of ganglion cell dendrites and axons occurs prior to vascular defects in type 1 diabetic mice [26]. These changes are limited to large and medium ON-type ganglion cells and do not occur in any class of OFF-type ganglion cells. To our knowledge, there is no information regarding the changes in electrophysiological properties of retinal ganglion cells during the early stage of type 1 diabetes.

Retinal ganglion cells intrinsically generate spontaneous spikes (action potentials) that are involved in regulating communication between the retina and the rest of the brain [27]. The spontaneous activity of ON-type ganglion cells is maintained by presynaptic inputs [28]. These inputs include excitatory glutamatergic input from ON-type bipolar cells and inhibitory GABAergic and glycinergic inputs from amacrine cells [29].In this study, we sought to determine whether and how the spontaneous activity of retinal ganglion cells is altered in streptozotocin (STZ)-induced diabetic mice, a model of human type 1-like diabetes [30], [31]. We found that ON-type retinal ganglion cells were hyperactive in diabetes and that the hyperactivity appeared to be predominantly mediated by a diabetes-induced reduction in inhibitory amacrine cell signaling.

## Materials and Methods

### Animals

C57BL/6 male mice aged 7- to 8-weeks-old purchased from SLAC Laboratory Animal Co. Ltd. (Shanghai, China) and The Jackson Laboratory (Bar Habor, ME, USA) were housed in 12:12-h light/dark. Use and handling of mice were in strict accordance with the ARVO Statement for the Use of Animals in Ophthalmic and Vision Research and were approved by Institutional Animal Care and Use Committees of Fudan University and Oakland University. The mice were randomly assigned to diabetic or control groups. After being fasted for 4 h, mice in the diabetic group received an intraperitoneal injection of 65 mg/kg STZ(Sigma-Aldrich Co., St. Louis, MO, USA) for three consecutive days. STZ was dissolved in sodium citrate buffer (1%, pH 4.2). Control animals received injections of an equal volume of citrate buffer. Blood glucose concentrations were measured using a glucometer (Accu-Chek Advantage, Roche, Germany) at several time points: prior to injection, after 7 days, and at 1, 2, and 3 months after the initial injection. Animals with fasting blood glucose concentrations higher than 11.1 mM were selected for experiments [32], [33].

### Tissue preparation

After being dark adapted for 2–4 h, mice were sacrificed by cervical dislocation. Eyes were immediately enucleated under dim red illumination and placed in oxygenated extracellular medium at room temperature. The extracellular solution contained (in mM): 125 NaCl, 2.5KCl, 1 MgSO_4_, 2 CaCl_2_, 1.25 NaH_2_PO_4_, 20 glucose, and 26 NaHCO_3_, bubbled with 95% O_2_ and 5% CO_2_. The eye was opened by an encircling cut along the ora serrata and the retina was separated from the sclera. The flat-mount retina was placed photoreceptor-side down in a custom-made recording chamber. The chamber was mounted on the stage of an upright microscope (Axioskop 2 FS Mot, Carl Zeiss, Jena, Germany) equipped with differential interference contrast (DIC) optics. Oxygenated medium was continuously perfused into the recording chamber at a flow rate of 1.5–2 ml/min with a peristaltic pump (Rainin Instrument Co. Inc., Oakland, CA, USA) and the superfusate was kept at 30–34°C by a temperature-control unit (TC-324, Warner Instruments, Hamden, CT, USA).

### Electrophysiological recordings

Retinas were maintained in darkness for at least 1 h prior to recording. Cells and recording pipettes were viewed on a video monitor coupled to a CCD camera (C2400-79, Hamamatsu, Hamamatsu City, Shizuoka Prefecture, Japan) mounted on the microscope. The pipette was advanced to an alpha-type ganglion cell in the ganglion cell layer (see Results for details) using visual control under infrared illumination. Gentle suction was applied to establish a loose-patch configuration with a seal resistance of 15–25 MΩ. Recording pipettes were prepared from thick-walled borosilicate filament glass tubing (Sutter, Novato, CA, USA) using a P-97 Flaming/Brown micropipette puller (Sutter) and filled with a solution containing (in mM) 150 NaCl and 10 HEPES (pH 7.5). The resistance of pipettes filled with this solution was 3–4 MΩ. The pipettes were mounted on a motor-driven micromanipulator (MP-285, Sutter) and connected to a patch-clamp amplifier (EPC-9, Heka, Lambrecht/Pfalz, Germany). Data were collected using Pulse 8 software (Heka). Prior to the experiments, electrical activity of ganglion cells was detected under voltage-clamp mode with a holding potential of 0 mV as well as in current-clamp mode with a holding current of 0 pA. It was found that the cell spontaneous firing rate recorded under the voltage clamp mode (9.03±2.99 Hz) was almost identical with that in the current-clamp mode (9.00±2.95 Hz, P>0.05, paired Student's t-test, n = 6). Therefore, all the experiments described in the present study were conducted under the voltage-clamp mode. Currents recorded were low-pass filtered at 2.9 kHz and sampled at 5 kHz.

### Light stimulation

Light stimuli were generated using an LED (λ = 525 nm). Full-field illumination was delivered from the LED to the retina through the microscope condenser. The LED was controlled by Pulse software and light intensity was adjusted by varying the output voltage from the software. Photon fluxes on the surface of the superfusion chamber were measured with a linear/log optometer (S350, UDT Instruments, San Diego, CA, USA). The stimulus intensity varied from 3.36×10^8^ to 3.36×10^11^ photons/cm^2^/s.

### *In situ* detection of DNA fragmentation by TUNEL assay

TUNEL-FITC labeling was performed in isolated retinas using the DeadEnd™ Fluorometric TUNEL Detection System kit (Promega, Madison, WI, USA), following the manufacturer's instructions. The retinas were counter-stained with DAPI (Vector Lab, Burlingame, CA, USA) to reveal cell nuclei. To distinguish TUNEL-positive structures from autofluorescence, retinas were sequentially examined with rhodamine and FITC filters; autofluorescent structures were visible under both filters, whereas TUNEL-positive cells were only detectable with the FITC filter. The retinas were further examined with the UV filter (for DAPI) to confirm the co-localization of TUNEL signals with cell nuclei. The total number of TUNEL-positive cells of each retina, detected at different focal planes, were recorded using a computer-based Neurolucida system (Microbrightfield, Williston, VT, USA) with a 20× objective on an Olympus microscope (Olympus Corporation, Tokyo, Japan). 4 to 6 animals were used for each group.

### Immunocytochemistry

Mice were anesthetized and the retinas were removed and fixed with 4% paraformaldehyde. Retina whole-mounts were blocked with 1% bovine serum albumin and 0.3% Triton X-100 in 0.1 M PBS for 2 h, and then incubated with a mouse monoclonal antibody against a non-phosphorylated epitope neurofilament H (SMI-32, 1∶5000, CovanceInc, Seattle, WA, USA) for 36–48 h. After rinsing, a secondary incubation containing Alexa Fluor 488-conjugated donkey anti-mouse IgG (1∶500 Molecular Probes, Eugene, OR, USA) was performed for 2 h. Samples were sealed with a coverslip using Vectashield (Vector Lab) and then visualized using conventional fluorescence microscopy. SMI-32 antibody labels somata and dendrites of α-type ganglion cells as well as some small ganglion cells [34], [35]. SMI-32-labeled cells present in this paper were large α-type ganglion cells counted from the peripheral retinas of mice.

### Chemicals

L-(+)-2-amino-4-phosphonobutyric acid (L-AP4, group III metabotropic glutamate receptor agonist), D-(−)-2-amino-5-phosphonopentanoic acid (D-AP5, NMDA receptor antagonist), 6-cyano-7-nitroquinoxaline-2,3-dione (CNQX, AMPA/kainate receptor antagonist), and (-)-Bicucullinemethiodide (GABA_A_ receptor antagonist) were purchased from Tocris (Tocris Bioscience, Bristol, United Kingdom). Strychnine hydrochloride (glycine receptor antagonist) was obtained from Sigma (Sigma-Aldrich Co.). All drugs were prepared as concentrated stock solutions, stored at −20°C, and freshly diluted to working concentrations in extracellular medium.

### Data analyses

Data were analyzed using the Pulsefit 8 (Heka), Clampfit 10 (Molecular Devices, Sunnyvale, CA, USA), OriginPro 7.0 (OriginLab Corp., Northampton, MA, USA) and SPSS 17 (IBM, Armonk, New York, USA) software packages. Firing rate and bursting activity were measured from 1-min recording events using the Event Detection program of the Clampfit 10 software. The following criteria were used to detect bursts using the ‘Poisson Surprise’ method of Clampfit 10: (1) at least three successive spikes within the burst; (2) burst onset initiated by two consecutive spikes with an inter-spike interval <80 ms; (3) burst terminated with two spikes having an inter-spike interval >160 ms [36]; (4) a Poisson Surprise value >1.5 [37].

The distribution normality of the data was determined using a One-Sample Kolmogorov-Smirnov test. When the data fitted a normal distribution, comparison between two independent groups was made by a Student's t-test. When the distribution was non-normal, a non-parametric Mann-Whitney U test was used. The levels of significance were set at P<0.05 (*), P<0.01 (**), and P<0.001 (***). Values of the normally distributed data are given as the mean ± SEM.

## Results

**Figure 1:**
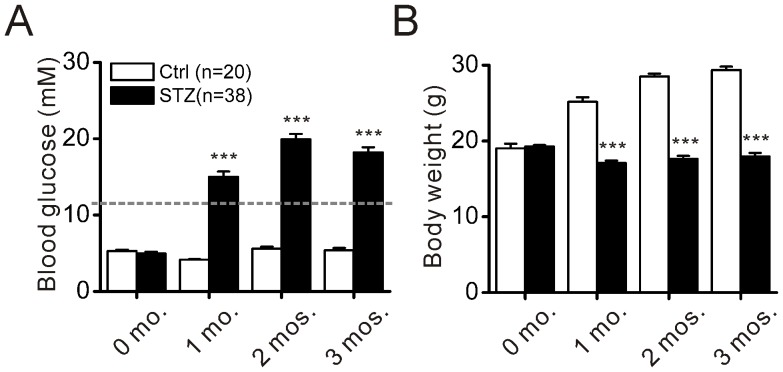
Blood glucose levels and body weights of control and STZ-induced diabetic mice. A. Blood glucose levels of control and diabetic mice are shown as a function of time after the induction of diabetes. Control and diabetic mice had similar blood glucose levels before injection of STZ. Control mice maintained blood glucose levels ∼5 mM over the course of the study, whereas blood glucose levels in the STZ-treated mice were increased by more than threefold at all three time points (*** P<0.001, Student's t-test).The grey dashed line indicates the blood glucose level of 11.1 mM. B. Age-matched control mice gained weight over the course of 3 months. In contrast, no weight increase was seen in diabetic mice. White bars show the age-matched control group (Ctrl); black bars indicate the STZ-treated group (STZ).

We aimed at determining whether and how diabetes influences electrical activity of retinal ganglion cells in mice. Diabetic mice were induced by intraperitoneal injection of STZ. As shown in Fig. 1A, blood glucose levels sharply increased from 4.95±0.23 mM prior to STZ injection to 14.99±0.72 mM the first month after injection and continued to increase until month 2 (19.92±0.70 mM). The elevated level persisted at month 3 (18.2±0.67 mM). No significant increase in body weight was observed over the course of 3 months (Fig. 1B). In contrast, during the same time period, age-matched control mice achieved normal weight gain (Fig. 1B) and had no significant increase in blood glucose levels (Fig. 1A).

**Figure 2:**
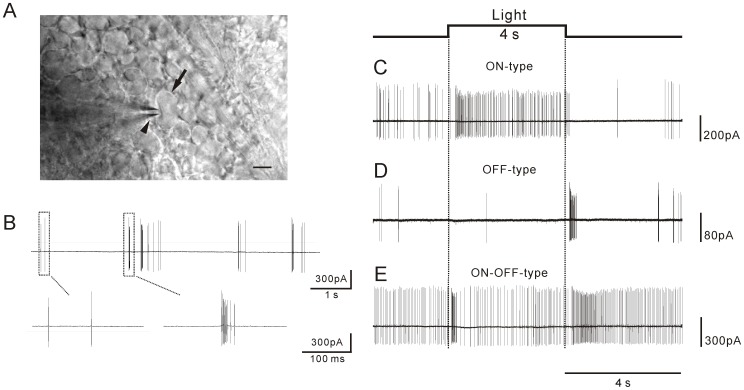
Spontaneous and light-evoked spike activity of retinal ganglion cells. A. A DIC image taken from the ganglion cell layer in a whole-mount mouse retina (scale bar indicates 10 µm). The arrow and arrowhead point to the soma of a representative α-type ganglion cell and the recording pipette, respectively. B. Spontaneous activity of an α-type ganglion cell. The cell exhibited a mixture of single spikes (left inset) and bursts (right inset). Typical recordings of representative ganglion cells showing ON (C), OFF (D), and ON-OFF (E) responses to 4-s full-field light stimuli (indicated by square wave above response traces; light intensity: 1.06×10^9^ photons/cm^2^/s).

**Figure 3:**
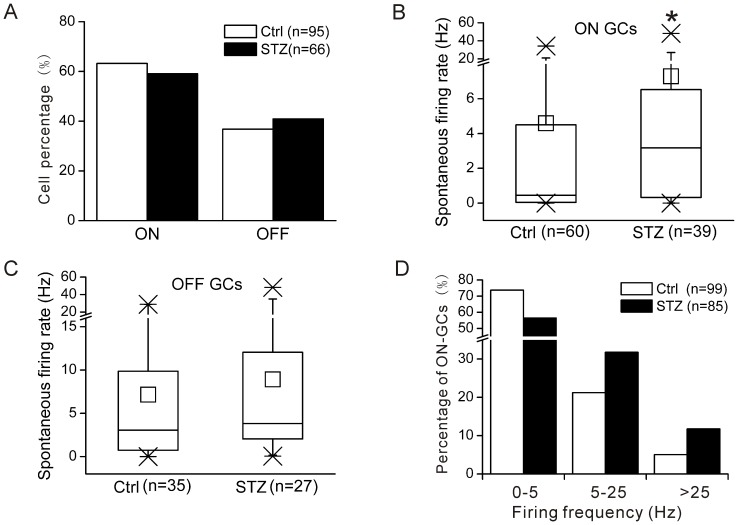
Spontaneous firing rates of ON-type ganglion cells were increased in diabetic mice. A. The percentages of ON- and OFF-type ganglion cells in both control and diabetic mice. B. Box and whisker charts illustrating the distribution of average spontaneous firing rates of ON-type ganglion cells in control and diabetic mice. In the charts, the middle line in the box represents the median, the box ends represent the first and third quartiles, whiskers indicate the 5% and 95% margins, and crosses indicate the data margins. In addition, the mean value is indicated by a small square. The diabetic ON-type ganglion cells showed a significant increase in spontaneous firing rate (*P<0.05, Mann-Whitney U test). C. Box and whisker charts illustrate the distribution of the average spontaneous firing rates of OFF-type ganglion cells in control and diabetic retinas. No difference was detected in spontaneous activity in OFF-type ganglion cells between control and diabetic groups (P>0.05). D. Histograms showing the percentage of all ON-type ganglion cells recorded as a function of firing frequency. The first band includes firing rates from 0–5 Hz, the middle band includes firing rates of 5–25 Hz, and the right band represents the firing rates >25 Hz. In diabetic mice, a considerable decrease was observed in the percentage of cells with an average firing frequency <5 Hz, but in the other two frequency bands (≥5 Hz) the percentage of diabetic cells were larger than the control group.

The diabetic (3 to 4 months of diabetes) and age-matched control mice were used for all experiments conducted below. The experiments were performed on dark-adapted flat-mount retinas. Electrical activity of ganglion cells was recorded from the retinas using a loose-patch extracellular recording technique. Mouse retinal ganglion cells have been classified into four groups (RG_A_ to RG_D_) based on their morphological parameters [38]. Here we chose cells from the RG_A_ group (corresponding to α-type ganglion cells) that have large somata (∼20 µm) for recording because they can be easily distinguished from other ganglion cells and displaced amacrine cells in the ganglion cell layer (Fig. 2A). The physiological properties of this cell class have been characterized and they exhibit ON or OFF responses to light [39]. Using the extracellular loose patch recording technique, we found that most of the cells recorded in control retinas exhibited spontaneous spiking activity with a mixture of single spikes and clusters of spikes (bursting) (Fig. 2B). A majority of the cells (∼60%, Fig. 3A) had increased spike frequency only at light onset (ON-type ganglion cells, Fig. 2C, 525-nm full-field stimuli), whereas ∼20% of the cells recorded had increased activity only at light offset (Fig. 2D, OFF-type ganglion cells). In addition, we observed that 20% of the cells recorded exhibited both ON and OFF responses (Fig. 2E). Since the full-field light stimuli may induce antagonistic surrounds in mouse ganglion cells, some OFF ganglion cells might show ON responses [40]. Therefore, these ON-OFF responsive cells were grouped into the OFF ganglion cell class for data analysis.

A total of 95 light-responsive cells were recorded from control retinas. Approximately 60% were ON-type and 40% were OFF-type (Fig. 3A). Under the same experimental condition, 66 light-responsive cells were recorded from diabetic retinas. The proportion of cell types in diabetes was similar to control retinas. Spontaneous firing rates of each cell were measured over a 1-min recording period. Data obtained from the same cell types were grouped for control and diabetic retinas, respectively. The grouped data from both control and diabetic ganglion cells exhibited a non-normal distribution (*P<0.05, One-Sample Kolmogorov-Smirnov test); therefore, box and whisker plots were introduced to quantitatively compare the spontaneous firing rates of cells between control and diabetic groups (Fig. 3B and 3C). The central values were expressed as medians and interquartile range (IQR) to avoid the misleading data that a highly skewed distribution could produce. Both median value and quartiles of ON-type ganglion cells were increased from control mice (0.47 Hz, median; 0.05–4.50 Hz, IQR; n = 60) to diabetic mice (3.17 Hz, median; 0.32–6.53 Hz, IQR; n = 39, Fig. 3B). A Mann-Whitney U test was performed, demonstrating a significant increase in the spontaneous spiking activity (*P<0.05, Fig. 3B). In addition, the spontaneous firing rate of OFF-type ganglion cells tended to increase from control retinas (3.05 Hz; 0.72–9.86 Hz; n = 35) to diabetic retinas (3.82 Hz; 2.04–12.05 Hz; n = 27, Fig. 3C); however, this increase was not significant (P>0.05, Mann-Whitney U test).

The above results demonstrated that the spontaneous activity of ON-type ganglion cells was significantly affected by diabetes, so we performed detailed analysis on this subtype. Figure 3D plots the percentages of ON-type ganglion cells as a function of firing frequency. The data clearly demonstrated that the percentage of cells with an average firing frequency <5 Hz decreased from control (73.74%) to diabetic retinas (56.47%). However, in the frequency band of 5–25 Hz, the percentage of cells in the diabetic group was larger than the control group. Furthermore, a dramatic increase was observed in cell percentage of the highest frequency band (>25 Hz; 11.76% in diabetes vs. 5.05% in control). It is noteworthy that ∼3.53% of ganglion cells in diabetic retinas exhibited an average frequency >35 Hz, yet no cells with this high firing rate were recorded in control retinas.

**Figure 4:**
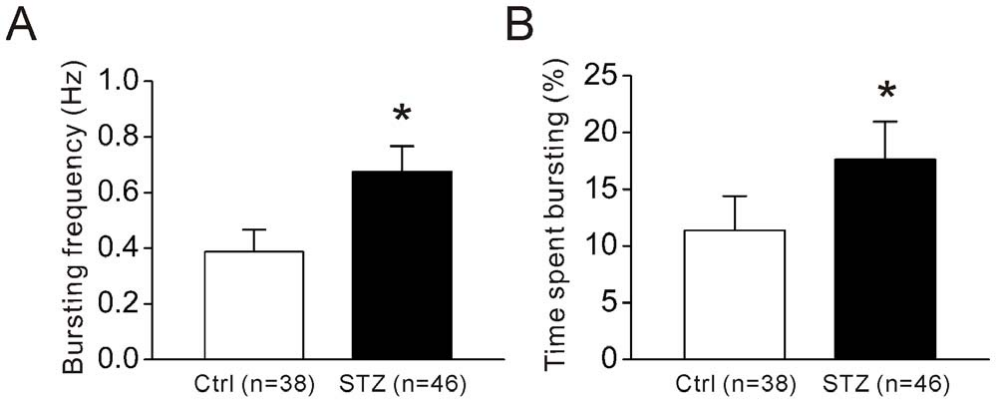
Enhanced bursting activity of diabetic ON-type ganglion cells. Histograms show that the bursting frequency (A) and time spent bursting (B) of ON-type ganglion cells were significantly increased in diabetic mice (*P<0.05, Student's t-test).

Since ganglion cells exhibit bursting activity, we further determined whether the bursting activity of ON-type ganglion cells was altered by diabetes. As shown in Figure 4A, the bursting frequency significantly increased from 0.39±0.08 Hz in control (n = 38) to 0.68±0.09 Hz in diabetes (n = 46, *P<0.05, Student's t-test). The percentage of time spent bursting was also significantly increased from 11.36±3.05% in control (n = 38) to 17.62±3.34% (n = 46) in diabetic mice (*P<0.05, Student's t-test; Fig. 4B). Furthermore, burst duration tended to increase, but the increase was not significant (215±35 ms in control vs. 321±26 ms in diabetes; P>0.05, Student's t-test). The results suggest that diabetes alters the firing pattern of ON-type ganglion cells.

**Figure 5:**
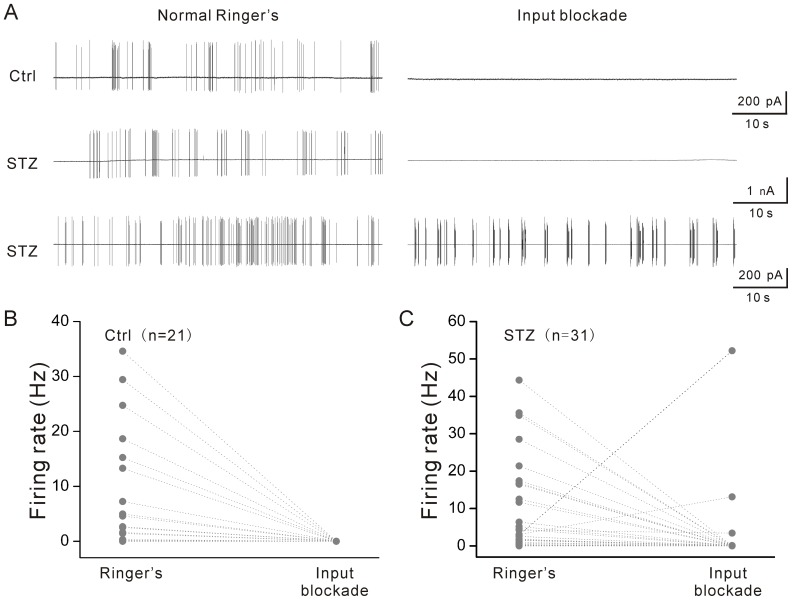
Effect of synaptic blockers on ON-type ganglion cell spontaneous activity. The cocktail of blockers contained (in μM): 50 L-AP4, 10 bicuculline, 10 strychnine, 50 D-AP5, and 10 CNQX. A. Representative traces show that spontaneous activity was completely eliminated in a control ON-type ganglion cell (upper panel) and a diabetic ON-type ganglion cell (middle panel), but was maintained to some extent in another diabetic ON-type ganglion cell (lower panel). Summarized data show that spontaneous firing rate of each ON-type cell was recorded before and after application of the synaptic blockers in control (B) and diabetic retinas (C). Grey circles show firing rate of each cell.

How does diabetes preferentially enhance ON-type ganglion cell spontaneous activity? It has been previously reported that spontaneous activity of ON-type ganglion cells is maintained by presynaptic input [28]. Indeed, we found that a cocktail of synaptic blockers (50 µM D-AP5, 10 µM CNQX, 10 µM bicuculline, and 10 µM strychnine) that block glutamatergic, GABA_A_, and glycinergic inputs to ganglion cells and along with 50 µM L-AP4 blocking the ON pathway, completely eliminated the spontaneous activity of all cells tested (n = 21) in control retinas (Fig. 5B). Figure 5A (upper panel) illustrates an example of such a cell. It is worth noting that we biasedly selected the 21 control cells (Fig. 5B) that had relatively higher spontaneous spiking frequency in order to elucidate the presynaptic mechanism. The same strategy was also used to select 31 ON-type cells from diabetic retinas (Fig. 5C). Because of the bias, the significant difference between the spontaneous firing rates of the control cells versus the diabetic cells shown in Figure 3B was not expected to be seen between Fig. 5B and Fig. 5C. Figure 5C demonstrates that, in diabetic retinas, the cocktail of synaptic blockers completely suppressed the spontaneous spiking activity in 27 out of 31 ON-type ganglion cells. One typical recording is illustrated in Fig. 5A (middle panel), indicating that the spontaneous activity in the majority of diabetic ON-type ganglion cells is likely to be maintained by the presynaptic inputs. Interestingly, 4 out of 31 cells recorded retained their spontaneous activity to some extent in the presence of the synaptic blocker cocktail (Fig. 5A, lower panel; Fig. 5C). The result suggests that diabetes likely enhances synaptic input to ON-type ganglion cells which is not completely blocked by the synaptic blockers we used in the present study.

**Figure 6:**
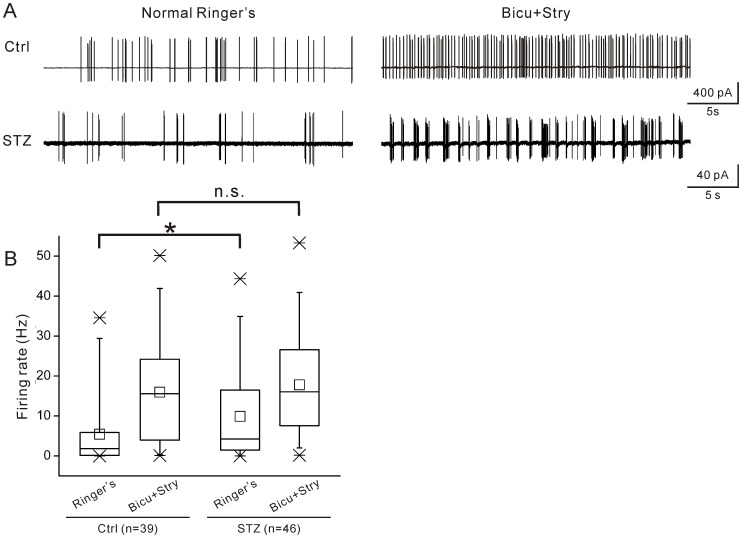
Suppression of inhibitory inputs to ON-type ganglion cells during diabetes. A. Typical traces of spontaneous activity show the effects of an inhibitory input blockade using bicuculline and strychnine on a control ON-type ganglion cell (upper panel) and a diabetic ON-type ganglion cell (bottom panel). B. Box and whisker plots show the distribution of average spontaneous firing rates of ON-type ganglion cells in control and diabetic mice before and after inhibitory input blockade. The difference in spontaneous firing rate between normal and diabetic ON-type ganglion cells in normal Ringer's solution (*P<0.05, Mann-Whitney U test) was eliminated in the presence of bicuculline and strychnine (P>0.05).

One possibility for the enhancement could be that diabetes attenuates inhibitory inputs from amacrine cells to ganglion cells; this disinhibition would then increase retinal ganglion cell spontaneous activity. To test this possibility, ON-type ganglion cell spontaneous activity in control and diabetic retinas was examined in the absence and presence of the GABA_A_ receptor blocker bicuculline (10 µM) and glycine receptor blocker strychnine (10 µM). Similar to the results shown in Figure 3B, without bicuculline and strychnine, the firing rate of cells in diabetic retinas was significantly higher than in control retinas (Fig. 6B). In the presence of bicuculline and strychnine, the median and quartiles of spontaneous firing rates were increased in both control (15.58 Hz, 3.59–24.20 Hz) and diabetic retinas(16.03 Hz, 7.55–26.62 Hz) (Fig. 6A, 6B). However, the significant difference between the control and diabetic groups observed in the absence of bicuculline and strychnine was not seen in the presence of these receptor antagonists (P>0.05, Mann-Whitney U test; Fig. 6B). These results suggest that diabetes appears to reduce the activity of GABA_A_ and glycine receptors and in turn increases ganglion cell firing rate.

Another possibility could be that ON-type ganglion cells may undergo apoptosis during diabetes which alters their electrical activity. We therefore examined apoptotic cells in whole-mount retinas by performing TUNEL staining analysis 3 months after diabetes induction. No apoptotic cells were observed in the ganglion cell layer of diabetic retinas (n = 6), suggesting that the observed alterations in ON-type retinal ganglion cell physiology are unlikely due to apoptosis. In addition, we detected an average of 4.83±0.82 TUNEL-positive cells per retina (n = 6) in other retinal layers. This number was similar to that observed in control retinas (4.00±0.82, n = 4; P>0.05, Student's t-test), indicating that our 3 months diabetic animal model did not have detectable diabetes-induced apoptosis in the retina. We also examined the number of α-type ganglion cells in diabetic and control retinas by marking the cells with SMI-32 antibody. No significant change was found in the cell numbers between diabetic (33.1±8.2 cells/mm^2^, 5 mice) and control retinas (32.5±9.2 cells/mm^2^, 5 mice, P>0.05, Student's t-test), suggesting that α-type ganglion cells likely remain intact during 3 months of diabetes.

## Discussion

### Functional alterations of retinal ganglion cells in early diabetes

A significant finding of the present work is the considerable increase in both spontaneous firing frequency and bursting activity of ON-type ganglion cells during an early stage of diabetes which was not detectable in OFF-type ganglion cells. This is the first characterization of ganglion cell electrical activity in a type 1 diabetic animal model. Our results and data from other investigators suggest that the ON signaling pathway in the retina could be preferentially affected by diabetes. In Ins2^Akita^ mice that spontaneously develop type 1-like diabetes, structural remodeling of dendrites was observed in large and medium ON-type ganglion cells, but not in OFF-type cells [26]. More recently, Xiao *et al*. (2012) demonstrated a reduction in receptive field size and elevation in luminance threshold in ON-type ganglion cells of db/db mice, a popular genetic model of type 2 diabetes [41].

The fact that ganglion cells spontaneously fire action potential implies that visual signals are transmitted to the visual centers by modulation of ever-present background noise [27], [42]. The increase in the background noise provided by ON-type ganglion cells appears to reduce the signal-to-noise ratio in the ON pathway. It is of interest that in type 1 diabetic patients, the pattern electroretinogram (PERG), a reflection of ganglion cell activity, was reported to show a significant decrease in amplitude [43], [44], implying a reduced visual signal. Concurrent increase in background noise and decrease in signal could further reduce the signal-to-noise ratio.

Such a functional alteration of the ON pathway may lead to an imbalance between the ON and OFF pathways, thus causing visual defects [7], [20], [45]–[47].

The hyperactivity of ON-type ganglion cells may also cause their apoptosis. A straightforward explanation could be that the elevated action potentials likely increase calcium influx, thereby inducing apoptosis of the ganglion cells. Available data concerning whether diabetes causes ganglion cell death in mice now seem inconsistent. Two research groups have reported that there was a significant loss of ganglion cells after only 6 weeks [24] or 10 weeks [19] of diabetes. However, other investigators found only a transient increase in TUNEL-positive cells after 2 weeks of diabetes and no ganglion cell loss was detected afterward and even after 1 year of diabetes in mice [48], [49]. Our data also demonstrated no significant increase in the number of TUNEL-positive cells and no α-type ganglion cell loss in 3-month-old diabetic retinas. The results suggest that the physiological alterations of ON-type ganglion cells observed in the present study are unlikely due to apoptosis; instead, persistent elevation of ganglion cell spiking activity during the early stage of diabetes may slowly increase intracellular Ca^2+^ and eventually lead to cell apoptosis during the late stage of the disease.

### Mechanisms underlying increased spontaneous activity of ON-type ganglion cells

The spontaneous activity of ganglion cells could be generated intrinsically and/or driven by input from presynaptic cells [28]. As shown in Figure 5, blockade of excitatory and inhibitory inputs abolished all spontaneous discharge of ON-type ganglion cells in control retinas; this finding suggests that these cells are unlikely to intrinsically generate spontaneous discharges in intact retinas, but that the spontaneous firing was driven by presynaptic inputs. This result is consistent with a previous study which was conducted in a normal light-adapted retina [28]. Similarly, the synaptic forces that maintain spontaneous spiking activity of ON-type ganglion cells likely remained intact in diabetic retinas because the majority of cells tested (27 out of 31) lost their spontaneous discharges after the application of synaptic blockers. In some cells (12.90%), however, the synaptic inputs appear enhanced by diabetes because their spontaneous discharges were preserved to some degree in the presence of the synaptic blockers. It should be indicated that our conclusion does not rule out the possibility that diabetes alters intrinsic membrane properties of ganglion cells which are responsible for the generation of intrinsic spontaneous activity. However this seems unlikely because we did not detect any significant change in spontaneous spiking activity in OFF-type ganglion cells. The OFF-type cell spontaneous activity has been reported to be primarily mediated by intrinsic activity [28]. This might be the reason that diabetes influences ON-type ganglion cells but not OFF-type ganglion cells at this early disease stage.

It is likely that there is profound interplay between spontaneous discharges driven by excitatory and inhibitory inputs and that the spontaneous activity of ON-type ganglion cells (when excitatory and inhibitory inputs are kept intact) is a result of such interplay. In agreement with previous work [29], the spontaneous firing rate was remarkably increased when GABA_A_ and glycinergic inputs were suppressed (Fig. 6). An interesting finding was that the difference in spontaneous spiking was eliminated when GABA_A_ and glycinergic signal transmission was blocked by the GABA_A_ and glycine receptor blockers (Fig. 6). This finding strongly suggests that the increase in spontaneous discharges of ON-type ganglion cells may be largely due to an attenuated suppression of GABA_A_ receptor and glycinergic signaling from amacrine cells on the spontaneous discharges driven by excitatory input from bipolar cells, although we cannot rule out an involvement of GABA_B_ and GABA_C_ receptors. Indeed, oscillatory potentials of the flash electroretinogram, which likely reflects synaptic activity between neurons in the inner retina, are either reduced or delayed in diabetic patients [7], [45] and in experimental diabetes, even at an early stage of diabetic retinopathy [20], [46], [47], [50]–[52]. Although the origin of oscillatory potentials is still unclear, there is evidence suggesting that GABAergic and glycinergic amacrine cells may contribute to the generation of the responses [53]. Whether the excitatory input-driven spontaneous activity of ON-type ganglion cells is directly enhanced by diabetes remains under investigation. These excitatory inputs include but are not limited to glutamatergic, nicotinic receptor, and substance P signaling [28], [54]–[57].

Bursting activity of ON-type ganglion cells was also altered by diabetes. In particular, both bursting frequency and time spent bursting were significantly increased in diabetic retinas. Similar phenomena were reported in retinal ganglion cells of the degenerating mouse retina [58]–[60]. It has recently been suggested that an electrically-coupled network of ON cone bipolar/AII amacrine cells constitutes an intrinsic oscillator in photoreceptor-degenerate retinas that is likely mediating increased bursting activity of ganglion cells [60]. Whether the increased bursting activity of ganglion cells in diabetes is mediated by a similar mechanism remains an open question for future studies.

In summary, the present study provides the first-ever characterization of retinal ganglion cell electrical activity in a type 1 diabetic mouse model. Our results indicate that diabetes preferentially increases the spontaneous firing frequency and bursting activity of ON-type ganglion cells. This ganglion cell hyperactivity appears to result from reduced presynaptic inhibitory signaling, which likely leads to a dysfunction in visual signal processing during the early stage of diabetes as well as progressive retinal cell apoptosis during the late stage of the disease.
